# Relationship of diffuse myocardial fibrosis to body composition: the Multi-Ethnic Study of Atheroscelerosis (MESA)

**DOI:** 10.1186/1532-429X-15-S1-O109

**Published:** 2013-01-30

**Authors:** Songtao Liu, Chia-Ying Liu, Jing Han, Evrim B Turkbey, Yuan Chang Liu, Diane Bild, Andrew E Arai, Robyn McClelland, W Gregory Hundley, Antoinette S Gomes, Russell Tracy, Richard Kronmal, Joao A Lima, David A Bluemke

**Affiliations:** 1 Radiology and Imaging Sciences, NIH Clinical Center, Bethesda, MD, USA; 2 Molecular Biomedical Imaging Laboratory, National Institute of Biomedical Imaging and Bioengineering, Bethesda, MD, USA; 3 Johns Hopkins Hospital, Baltimore, MD, USA; 4 U.S. Food and Drug Administration, Rockville, MD, USA; 5 National Heart Lung and Blood Institute, Bethesda, MD, USA; 6 University of Washington, Seattle, WA, USA; 7 Wake Forest University, Winston-Salem, NC, USA; 8 University of California Los Angeles, Los Angeles, CA, USA; 9 University of Vermont, Burlington, VT, USA

## Background

Patients who are overweight and obese have a better clinical prognosis than their lean counterparts, termed the "obesity paradox". The relationship of body composition to myocardial tissue composition however is largely unknown. The purpose of this study was to evaluate the relationship of body composition to diffuse myocardial fibrosis (DMF) as assessed by cardiac magnetic resonance (CMR) T1 mapping.

## Methods

A total of 608 participants (mean age 67.9 ± 8.8 years, 47.2% men) in the Multi-Ethnic Study of Atherosclerosis (MESA) who were free of clinically apparent cardiovascular disease at baseline enrollment underwent CMR to assess LV size and function as well as measures of body composition (body mass index, BMI), and cardiovascular risk factors. Gadolinium contrast (Magnevist, 0.15 mmol/kg) was administrated and single slice T1 mapping was performed at the mid-ventricular level before and after (12 and 25 minutes) using a Modified Look Locker Inversion Recovery (MOLLI) sequence. Study subjects with LGE scar were excluded. Extracellular volume fraction (ECV) was derived by adjusting partition coefficient with hematocrit. The subjects were divided into six groups according to WHO BMI classification. Linear regression analysis was used to evaluate the association of BMI categories with ECV. Sequential models were developed. Model 1 was adjusted for demographic information (age, gender and ethnicity); Model 2 included model 1 variables plus traditional risk factors (smoking, diabetes and blood pressure); Model 3 included model 2 variables plus height.

## Results

**Figure 1:**
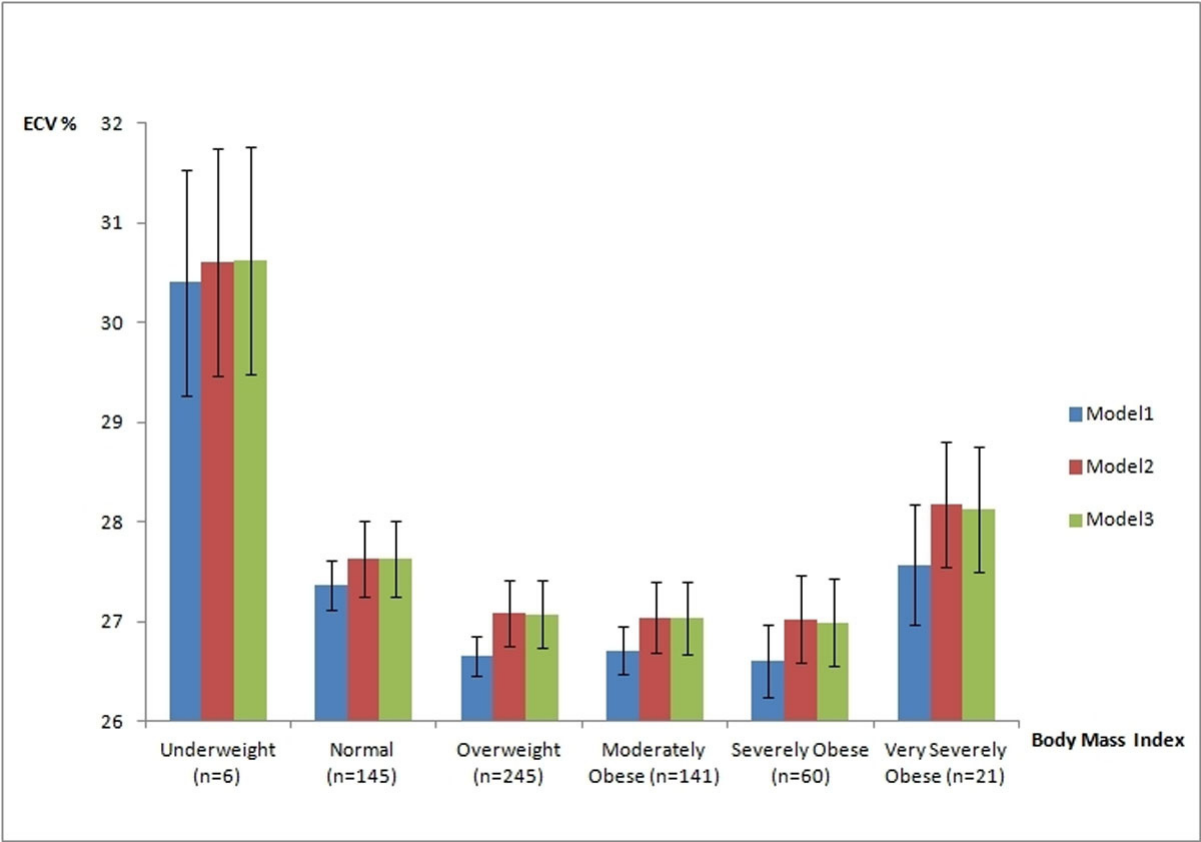
Association of the spectrum of body mass index with extracellular volume fraction, with adjustment for demographic data, traditional cardiovascular risk factors and height. Model 1: adjusted for demographic information (age, gender and ethnicity); Model 2: adjusted for model 1 variables and traditional risk factors (smoking, diabetes and blood pressure); Model 3: adjusted for model 2 variables plus height.

The average BMI and ECV were 28.9 ±5.4, and 0.27±0.03, respectively. ECV was significantly associated with BMI categories in all three models (ECV vs. BMI, p=0.006, model 3) (Figure 1). Age, gender, smoking, diabetes and hypertension were also significantly associated with ECV. ECV was not associated with ethnicity and height. The underweight group (BMI <18.5) had a significantly greater ECV compared with BMI between 18.5 and 40 (p=0.003). The very severely obese group (BMI >40) had a trend towards higher ECV compared with BMI between 18.5 and 40 (p=0.09) (Table 1).

**Table 1:** Associations of Body Mass Index categories with extracellular volume fraction

	Body Mass Index		
	
	<18.5 (Underweight) N=6 (1%)	18.5-40 N=581(95.5%)	>40 (Very severely obese) N=21(3.5%)
Model 1*	3.53 (1.32 to 5.77); 0.002	Reference	0.75 (-0.45 to 1.96); 0.22

Model 2+	3.35 (1.17 to 5.54); 0.003	Reference	1.04(-0.17 to 2.24); 0.09

Model 3#	3.37 (1.18 to 5.55); 0.003	Reference	1.02 (-0.19 to 2.23); 0.09

Values are β (95% confidence interval), p value. *Adjusted for age, sex, and race. +Adjusted for Model 1 variables plus traditional risk factors (hypertension, smoking and diabetes). #Adjusted for Model 2 variables plus height.

## Conclusions

In a multiethnic population, extremes of body composition had higher level of diffuse myocardial fibrosis detected by CMR, especially for underweight study subjects.

## Funding

National Institutes of Health Intramural Program.

